# Low-dose 10-day decitabine plus venetoclax induced rapid MRD negativity and durable remission in De Novo AML

**DOI:** 10.1007/s00277-026-07222-7

**Published:** 2026-08-19

**Authors:** Junqing Wu, Xueping Luo, Xiaoyu Liu, Jingwen Li, Shan Fu, He Huang, Weiyan Zheng

**Affiliations:** 1 Bone Marrow Transplantation Center, the First Affiliated Hospital, Zhejiang University School of Medicine, Hangzhou, China; 2 Liangzhu Laboratory, Zhejiang University Medical Center, Hangzhou, China; 3 Institute of Hematology, Zhejiang University, Hangzhou, China; 4 Zhejiang Province Engineering Laboratory for Stem Cell and Immunity Therapy, Hangzhou, China

**Keywords:** Acute myeloid leukemia, Decitabine, Venetoclax, Low‑dose

## Abstract

The combination of hypomethylating agents and venetoclax (VEN) has emerged as a new standard of care for elderly or unfit patients. In this study, we conducted a retrospective analysis of 37 patients with acute myeloid leukemia (AML) to investigate the efficacy and safety of a low‑dose 10‑day decitabine (DEC) combined with venetoclax (VEN) regimen (DEC 6 mg/m^2^ for 10 days and VEN for 14 days). The composite complete remission (CRc) rate was 78.4%, with 88.9% in favorable group and 75.0% in intermediate/adverse group according to ELN-2022 risk stratification. Among patients who achieved CRc, 79.3% patients attained MRD negativity after the first cycle. With a median follow-up of 40.0 months, the median overall survival (OS) was 36.1 months. The 36-month OS and EFS rates were 35.0% and 40.4%. Hematologic toxicities were the most common adverse events. The multivariate analysis showed that patients with ECOG < 3 and non-complex karyotype had significantly better OS. The regimen of low-dose 10-day DEC plus VEN induced rapid MRD negativity with good tolerability, along with high long-term survival in patient ineligible for intensive therapy.

**Supplementary Information:**

The online version contains supplementary material available at https://doi.org/10.1007/s00277-026-07222-7.

## Introduction

Acute myeloid leukemia (AML) is hematologic malignancies associated with poor prognosis [1, 2]. The five-year overall survival (OS) rate is below 10% for patients over 60 [3]. Therefore, active treatment is essential to improve survival rate [4].

The combination of hypomethylating agents (HMAs) and venetoclax (VEN) has emerged as a new standard of care for patients who are ineligible for intensive chemotherapy [5, 6]. In the VIALE-A trial, the composite complete remission (CRc) rate reached 73% in the HMA + VEN cohort [7]. Compared to intensive chemotherapy, the HMAs-VEN regimen exhibited a favorable safety profile, including a lower incidence of febrile neutropenia, fewer serious infections, and reduced reliance on supportive care [8].

As one of the HMAs, decitabine (DEC) has demonstrated notable efficacy. The CRc rate with DEC + VEN was reported as high as 89% among patients with AML under 60 years old, while another trial reported a rate of 74% [8, 9]. In elderly patients with AML, however, outcomes remain challenging. It was reported a 2-year OS rate of 25% in elderly AML patients treated with DEC [10].

The standard 5‑day DEC regimen of 20 mg/m^2^/day is often associated with prolonged bone marrow suppression and increased infection risk [11]. Recent evidence suggested that extending DEC exposure, for example, to 10 days per cycle rather than 5, may improve the objective response rate [12]. It prompted the exploration of low‑dose, prolonged DEC schedules. For instance, one study using DEC at 5.15–7.62 mg/m^2^/day for 10 days in combination with cytarabine and G‑CSF reported an objective response rate of 57.1% [13]. Similarly, another trial employed DEC at 6 mg/m^2^ for 10 days [14]. Aligned with these findings, we employed a regimen of DEC at a dose of 6 mg/m^2^ over a 10-day period.

Recent studies also indicated that while initial HMAs-VEN trials employed 21- to 28-day VEN schedules, shorter durations of 7–14 days might maintain similar efficacy with reduced toxicity [15–18]. Accordingly, the duration of the VEN in our regimen was defined as 14 days.

In this study, we retrospectively evaluated the efficacy and safety of a regimen consisting of low-dose DEC (6 mg/m^2^ for 10 days) combined with a shortened VEN exposure (14 days) in a cohort of elderly or unfit AML patients. We aimed to improve OS while reducing treatment-related adverse events.

## Methods

### Patients and regimen

Between April 2020 and May 2023, a total of 37 patients with AML were included in this retrospective analysis. Inclusion criteria were: 1) age ≥ 60 years, or 2) age < 60 years and classified as unfit or frail, making them ineligible for intensive chemotherapy. Among the AML patients, 24 were M0 or M2 (primitive phenotype), and 13 were M4 or M5 (monocytic/mixed phenotype). An additional four patients with IPSS-M high-risk myelodysplastic neoplasms (MDS) were enrolled. However, analyses were restricted to patients with AML. The regimen of low-dose DEC-VEN consisted of DEC (6 mg/m^2^ for 10 days) and VEN (100 mg d1, 200 mg d2, 400 mg d3-14, or equivalent dose if on concomitant azole). Each treatment cycle lasted 30–45 days, and the cycle length could be extended in patients with slow hematologic recovery. For patients maintained a CR state, the interval between two cycles could be prolonged up to 3 months. The regimen demonstrated favorable tolerability, with no dose reduction implemented in any patient. For patients who did not undergo HSCT, the total planned number of treatment cycles was 8–10. Additional treatment cycles could be administered at the patient's discretion. Following each treatment cycle, bone marrow (BM) aspiration was performed, and minimal residual disease (MRD) was assessed using flow cytometry.

### Outcomes

Complete remission (CR) was defined as BM blasts < 5%, accompanied by an absolute neutrophil count ≥ 1000/μL and a platelet count ≥ 100,000/μL. CR with incomplete hematologic recovery (CRi) met all CR criteria except for residual neutropenia (ANC < 1000/μL) or thrombocytopenia (platelet count < 100,000/μL). CRc included both CR and CRi. OS was calculated as the time in days from the date of diagnosis to death from any cause. Event-Free Survival (EFS) was defined as the time from diagnosis to the occurrence of any of the following events: disease progression, treatment failure, relapse, or death from any cause.

### Statistical analysis

Patient characteristics were summarized by percentage (frequency) for categorical variables and median (range) values for continuous variables. The statistical significance in differences between categorical variables was determined using the Chi-square or Fisher's exact test. OS and EFS were estimated using the Kaplan–Meier method and differences between groups evaluated using the log-rank test. Multivariate analyses were performed using Cox proportional hazards regression.

### Ethics approval and consent to participate

All procedures were conducted in compliance with relevant guidelines and regulations. Informed consent was obtained from all participants in accordance with the principles of the Declaration of Helsinki.

## Results

### Clinical characteristics

**Fig. 1:**
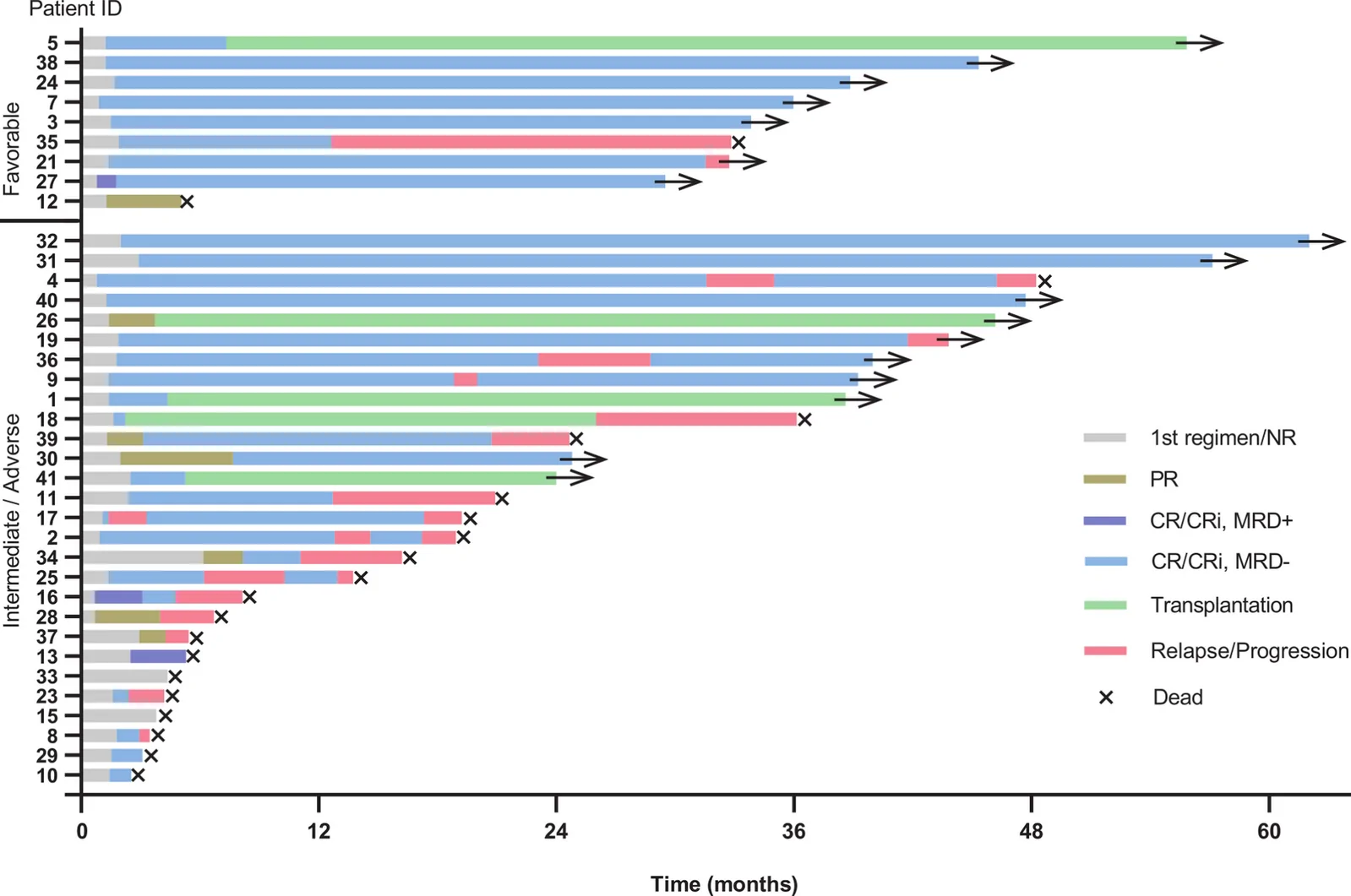
Swimmer plot of dynamic response assessment based on ELN-2022 risk stratification

Between April 2020 and May 2023, a total of 37 patients with AML were treated with the low-dose DEC-VEN regimen (Fig. 1A and Supplementary Fig. 1A). The median age was 66 years old. Patients under 60 years of age were deemed ineligible for intensive chemotherapy due to significant comorbidities. Based on the ELN 2022 risk stratification, 75.7% of patients were classified into the intermediate/adverse risk group. This proportion was 54.1% according to the ELN 2024 risk stratification. Five patients subsequently underwent allogeneic hematopoietic stem cell transplantation (allo-HSCT) (Table 1 and Supplementary Fig. 1B).

**Table 1:** Baseline demographic and clinical characteristics

Characteristic	*N* (%)
Age at diagnosis,median [range], y	66 [27–86]
Age group	
< 60	10 (27.0)
≥ 60	27 (73.0)
Sex	
Male	17(45.9)
Female	20 (54.1)
ECOG	
< 3	16 (43.2)
≥ 3	21 (56.8)
ELN-2022 risk stratification	
Favorable	9 (24.3)
Intermediate	8 (21.6)
Adverse	20 (54.1)
ELN-2024 risk stratification	
Favorable	17 (45.9)
Intermediate	15 (40.5)
Adverse	5 (13.5)
Cytogenetic Evaluation	
Normal karyotype	25 (67.6)
Complex karyotype	4 (10.8)
Others	8 (21.6)
Molecular Evaluation	
NPM1,CEBPA	10 (27.0)
FLT3-ITD	8 (21.6)
TP53	5 (13.5)
IDH1,IDH2, DNMT3A, TET2	16 (43.2)
KRAS, NRAS, PTPN11, CBL, NF1	18 (48.6)
SRSF2, SF3B1, U2AF1, ZRSR2, ASXL1	11 (29.7)
WBC count	
< 10 × 10E9/L	17(45.9)
≥ 10 × 10E9/L	20 (54.1)
BM blast	
< 50%	14(37.8)
≥ 50%	23 (62.2)
Received allogeneic HSCT	5 (13.5)

Additionally, four patients with high-risk MDS also received this regimen (Supplementary Fig. 1A). However, subsequent analyses focused on the 37 patients with AML.

### Efficacy

Overall response rates are summarized in Table 2. The CRc was achieved in 78.4% patients (29/37, [95CI, 64.5–92.3]). Of these, 70.3% patients (26/37, [95CI, 54.8–85.7]) attained CRc after the first cycle. According to ELN 2022 risk stratification, CRc rates were 88.9% (8/9, [95CI, 63.3–114.5]) in the favorable-risk group and 75.0% (21/28, [95CI, 57.9–92.1]) in the intermediate/adverse-risk groups. By immunophenotype, CRc was observed in 70.8% (17/24, [95CI, 51.2–90.4]) of patients with primitive phenotype and 92.3% (12/13, [95CI, 75.6–109.1]) of those with monocytic/mixed phenotype (Supplementary Table 1). While the favorable-risk group and the monocytic/mixed phenotype group showed higher CRc rates, the difference did not reach statistical significance.

**Table 2:** Response assessment

	Total (*N* = 37)		Intermediate & Adverse(*N* = 28)		Favorable (*N* = 9)	
Variable	*n*	%(95%CI)	*n*	%(95%CI)	*n*	%(95%CI)
CRc rate	29	78.4 (64.5–92.3)	21	75.0(57.9–92.1)	8	88.9(63.3–114.5)
CR	24	64.9 (48.7–81.0)	16	57.1(37.6–76.7)	8	88.9(63.3–114.5)
CRi	5	13.5 (2.0–25.1)	5	17.9(2.7–33.0)	0	0.0(0.0–0.0)
CRc after the first cycle	26	70.3 (54.8–85.7)	18	64.3(45.4–83.2)	8	88.9(63.3–114.5)
CR	23	62.2 (45.8–78.6)	15	53.6(33.9–73.3)	8	88.9(63.3–114.5)
CRi	3	8.1 (-1.1–17.3)	3	10.7(-1.5–22.9)	0	0.0(0.0–0.0)
MRD negativity after the first cycle	23	62.2 (45.8–78.6)	16	57.1(37.6–76.7)	7	77.8(43.9–111.7)
Partial remission after the first cycle	6	16.2 (3.8–28.7)	5	17.9(2.7–33.0)	1	11.1(-14.5–36.7)
No response after the first cycle	5	13.9 (2.0–25.8)	5	17.9(2.7–33.0)	0	0.0(0.0–0.0)

The MRD-negative rate after the first cycle was 62.2% (23/37, [95CI, 45.8–78.6]). Among those who achieved CRc, 79.3% (23/29, [95CI, 63.6–95.0]) patients attained MRD negativity after the first cycle.

**Fig. 2:**
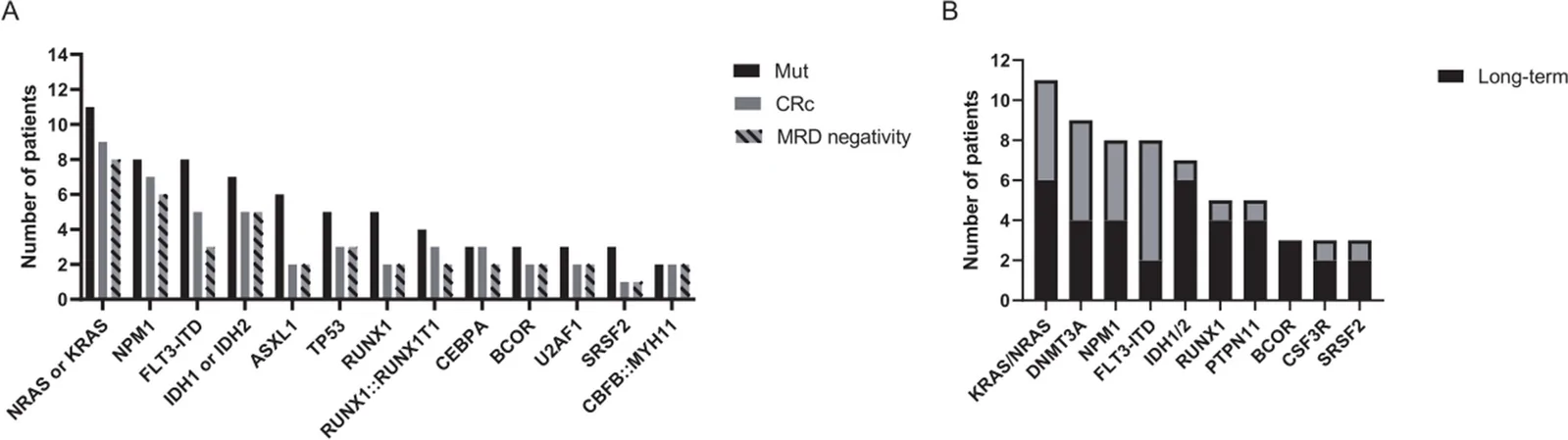
Gene distribution of patients who achieved CRc and MRD negativity after first cycle (**A**) and patients followed up for over 24 months (**B**)

After the first cycle, the numbers of patients with specific mutations and fusion genes who achieved CRc and MRD negativity are presented in Fig. 2A.

Patients received a median of 3 treatment cycles (range, 1–16). The median time to first CRc was 1.4 months (range, 0.7–3.3). The median duration of response (DOR) was 30.2 months (95% CI, 8.4–46.8) for patients achieving CRc, and 30.8 months (95% CI, 12.2.5–49.4) for patients achieving MRD negativity after first cycle.

### Survival

**Fig. 3:**
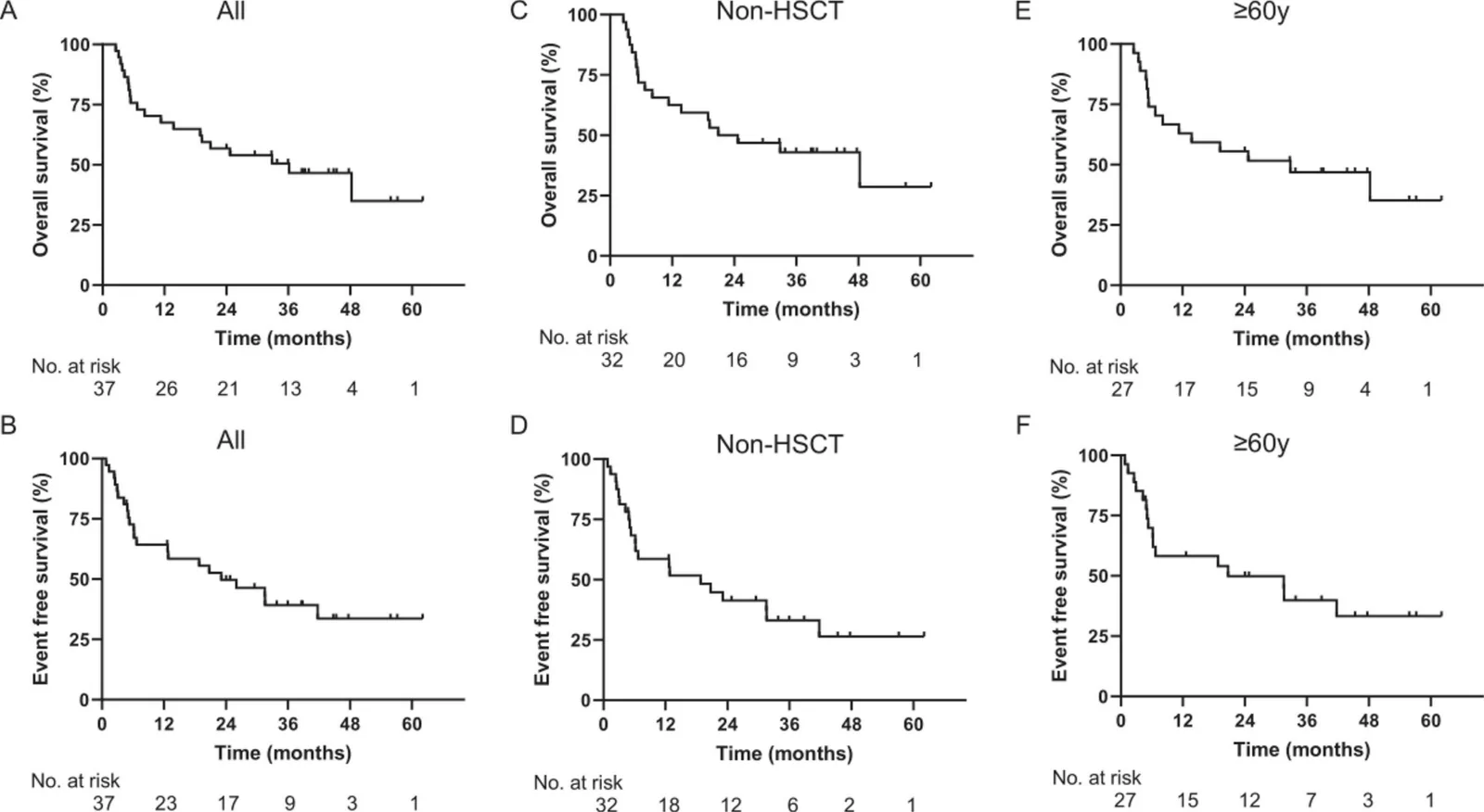
OS and EFS of all patients (**A**-**B**), patients did not receive allo-HSCT (**C**-**D**), and patients aged ≥ 60 years old (**E**–**F**)

The median follow-up was 40.0 months (range, 2.5–62.0). The median OS was 36.1 months (95% CI, 15.0–57.2) for all the patients, while 20.9 months (95% CI, 2.8–39.0) for patients who did not receive allo-HSCT. Among all patients, the 36‑month OS rate was 35.0% (95% CI, 11.5–58.5) (Fig. 3A and Table 3). In patients who received allo-HSCT, the 36-month OS rate was 75.0% (95% CI, 32.6–117.4), as compared to 42.9% (95% CI, 25.5–60.4) in those who did not (Fig. 3C). For those aged 60 years or older, the 36-month OS rate was 46.9% (95% CI, 27.6–66.2) (Fig. 3E). Notably, the mortality rate was 0% through both 30 and 60 days.

**Table 3:** The OS and EFS rates

		12 months		24 months		36 months	
Indicater	Patient	rate%	95%CI	rate%	95%CI	rate%	95%CI
OS	Total	64.9	49.5–80.2	56.8	40.8–72.7	35.0	11.5–58.5
	Non-HSCT	62.5	45.7–79.3	53.1	35.8–70.4	43.0	25.5–60.4
	≥ 60y	63.0	44.7–81.2	55.6	36.8–74.3	46.9	27.6–66.2
	Intermediate/adverse Intermediate/adverse	60.7	42.6–78.8	42.6	24.1–61.0	38.3	19.9–56.7
	(TP53^wt^)	65.2	45.8–84.7	43	22.6–22.6	/	/
EFS	Total	58.7	42.7–74.8	50.3	34.0–66.6	40.4	23.9–57.0
	Non-HSCT	52.1	34.5–69.6	42.3	24.9–59.7	34.6	17.4–51.8
	≥ 60y	58.2	39.3–77.1	50.4	31.2–69.6	41.3	21.8–60.7
	Intermediate/adverse Intermediate/adverse	48.8	29.9–67.6	37.5	19.2–55.8	28.1	10.4–45.9
	(TP53^wt^)	50.8	30.0–71.6	37.0	16.7–57.2	30.8	10.7–50.9

The median EFS was 26.0 months (95% CI, 8.7–43.3), while 18.8 months (95% CI, 0–40.9) for patients who did not receive allo-HSCT. The EFS rate at 36 months was 40.4% (95% CI, 23.9–57.0) (Fig. 3B and Table 3). Among non-HSCT patients, the 36-month EFS rate was 34.6% (95% CI, 17.4–51.8) (Fig. 3D). In patients aged ≥ 60 years, the 36-month EFS rate was 41.3% (95% CI, 21.8–60.7) (Fig. 3F).

**Fig. 4:**
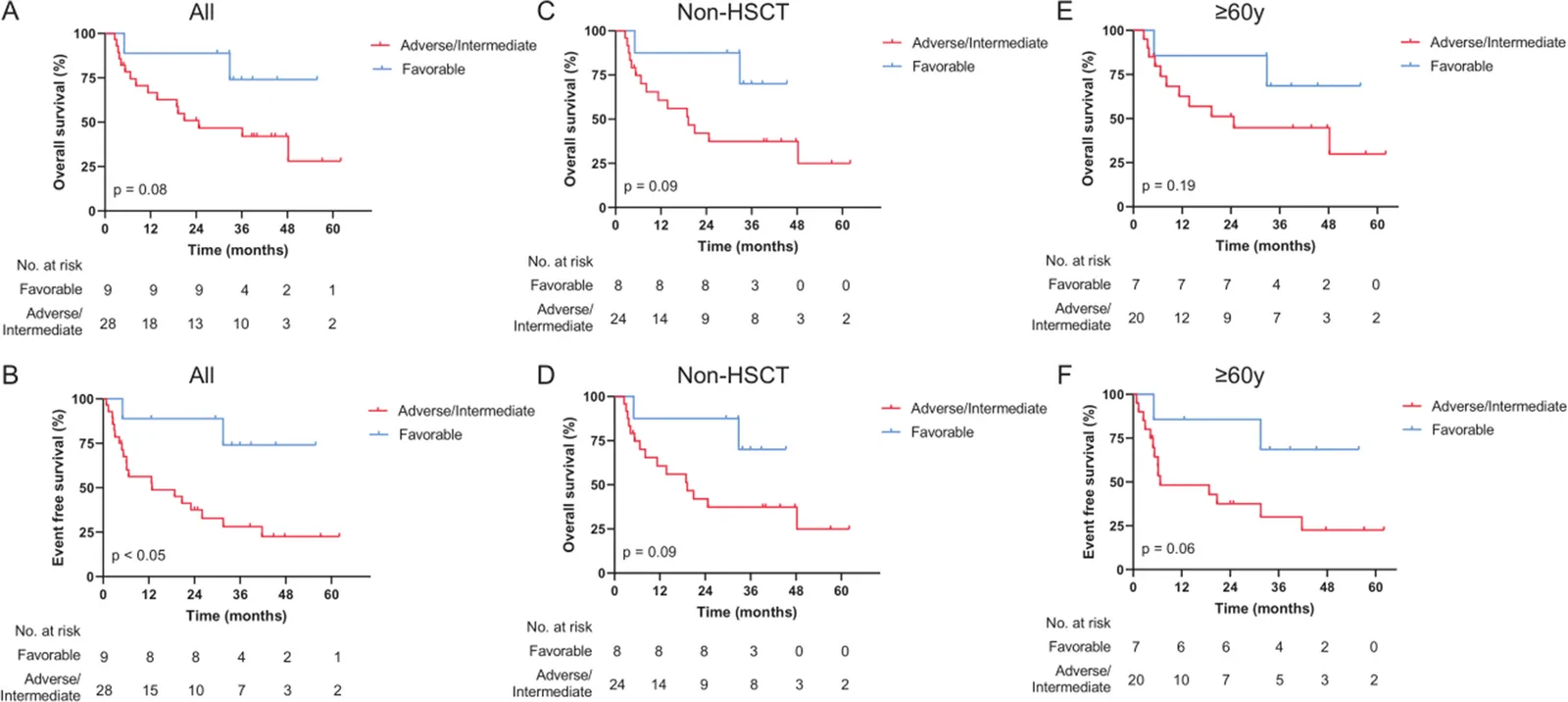
OS and EFS of all patients (**A**-**B**), patients did not receive allo-HSCT (**C**-**D**), and patients aged ≥ 60 years old (**E**–**F**) based on the ELN-2022 risk stratification

According to ELN 2022 risk stratification, patients in the favorable-risk group showed a trend toward better survival outcomes compared to those in the intermediate/adverse risk group. In the intermediate/adverse risk group, the 36-month OS and EFS rates were 38.3% (95% CI, 19.9–56.7) and 28.1% (95% CI, 10.4–45.9), respectively (Table 3, Fig. 4A–B). This trend was consistent among subgroups of patients who did not receive allo-HSCT and those aged ≥ 60 years (Fig. 4C–F). In contrast, no significant difference in OS was observed between risk groups when applying the ELN 2024 risk stratification (Supplementary Fig. 2). A potential explanation was the absence of IDH1/2 inhibitor therapy during that period.

### Molecular features

**Fig. 5:**
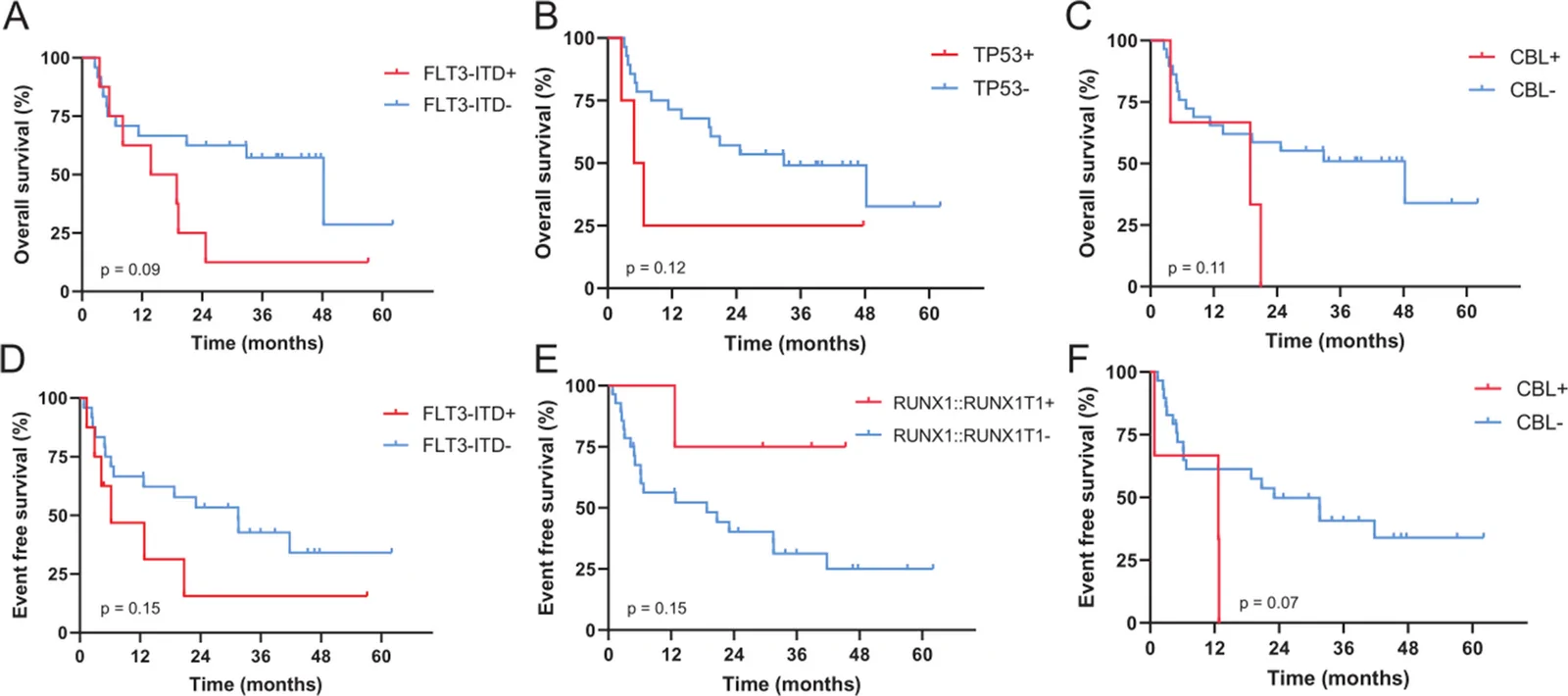
OS (**A**-**C**) and EFS (**D**-**F**) in patients according to specific mutation status

Although statistical significance was not reached, analyses revealed suggestive trends in the non-transplant patients: FLT3-ITD and CBL wild-type patients had improved OS and EFS, TP53 wild-type patients achieved better OS, and RUNX1::RUNX1T1-positive patients showed prolonged EFS (Fig. 5).

Compared with FLT3-ITD wild-type patients, FLT3-ITD-mutant patients had a median OS of 16.3 months (death range: 3.5–24.7; one patient censored at 57.1) vs. 48.2 months (death range: 2.5–48.2; censored follow-up: 24.8–62.0), and a median EFS of 6.2 months (event range: 1.4–20.7; censored follow-up: 4.7–57.1) vs. 31.5 months (event range: 0.8–41.7; censored follow-up: 12.6–62.0) (Fig. 5A and D). For TP53, median OS was 4.9 months (death range: 2.5–6.7; one patient censored at 47.7) in TP53-mutant patients vs. 32.8 months (death range: 3.1–48.2; censored follow-up: 24.8–62.0) in TP53 wild-type patients (Fig. 5B).

### Long-term survivor and patients received allo-HSCT

A total of 21 patients were followed up for over 24 months, including 14 who were followed for more than 36 months. In this long-term follow-up cohort, 90.5% (19/21) achieved CRc, and 9.5% (2/21) achieved PR before the third treatment cycle. The majority (76.2%, 16/21) did not undergo allo-HSCT, among whom 71.4% (10/14) received more than 5 cycles of the DEC + VEN regimen. The distribution of mutations was enriched in these long-term survivors was shown in Fig. 2B.

In our study, five patients who received allo-HSCT survived for more than 24 months. Of these, one patient with TP53 mutation died after 3 years since relapse, one survived following relapse, and four remained in CR. The survival time of the patient with TP53 mutation reached up to 36.1 months. As of the last follow-up, the longest survival time was 55.8 months. One patient presented with multiple gastrointestinal ulcers prior to allo-HSCT and has been followed for 24.0 months. Another patient received salvage allo-HSCT and has been followed for 44.7 months.

### Relapse patterns

We then analyzed the relapse patterns. Of the 29 patients who achieved CRc within one or two cycles, 53.3% (16/30) experienced relapse, with a median time to relapse of 12.8 months (range, 1.4–41.7). Three patients relapsed after completing ≥ 10 cycles of treatment, with relapse occurring at 2, 5, and 15 months following treatment cessation, respectively. One patient experienced relapse after 16 consecutive treatment cycles, and another relapsed post-HSCT. 12/30 patients were followed until death, and the median post-relapse survival was 6.8 months (range, 0.5–20.2), with a median OS of 19.1 months (range, 3.5–48.2).

Conversely, among the 7 patients who did not achieve CRc within one or two cycles, 71.4% (5/7) maintained PR or NR throughout follow-up until death, with a median OS of 5.1 months (range, 3.8–6.7). The remaining two patients were still alive: one received HSCT after maintaining PR, and the other achieved CR at cycle 4.

### Safety

As shown in Table 4, all patients who presented with grade ≤ 2 neutropenia at baseline developed grade 4 neutropenia during the first treatment cycle. The incidence of other grade ≥ 3 hematological adverse events was 73.3% (11/15) for thrombocytopenia and 88.9% (16/18) for anemia, respectively.

**Table 4:** Adverse events

Events, *n* (%)	Grades ≤ 2	Grades 3	Grades 4
Hematologic adverse events*			
Neutropenia	0	0	24 (100.0)
Thrombocytopenia	4 (26.7)	3 (20.0)	8 (53.3)
Anemia	2 (11.1)	6 (33.3)	10 (55.6)
Nonhematologic adverse events			
Fatigue	29 (78.4)	5 (13.5)	0
Decreased appetite	30 (81.1)	0	0
Constipation	16 (43.2)	0	0
Pneumonla	0	16 (43.2)	0
Hypocalcemia	10 (27.0)	0	0
Hypokalemia	9 (24.3)	0	0
ALT elevation	9 (24.3)	0	0
Sepsis	0	7 (18.9)	0
Insomnia	6 (16.2)	0	0
Nausea	5 (13.5)	0	0
Diarrhea	3 (8.1)	0	0
AST elevation	3 (8.1)	0	0
Hypotension	3 (8.1)	0	0

^*^Patients who entered the study with grade 3 neutropenia, thrombocytopenia or anemia were notevaluable for hematology related toxicities and thus were excluded from this analysis

During the first cycle, 12 patients remained platelet counts above 20 × 10⁹/L. Among 21 evaluable patients, the median time to platelet recovery (≥ 20 × 10⁹/L) was 20.0 days (range, 14–44). Twelve patients presented hemoglobin levels above 60 g/L. Among 22 evaluable patients, the median time to hemoglobin recovery (≥ 60 g/L) was 25.0 days (range, 8–44).

Among nonhematologic adverse events (AEs), fatigue was the most prevalent (91.9%, 34/37) of patients, followed by decreased appetite (81.1%, 30/37). Pneumonia developed in 43.2% (16/37) of patients, all of whom required intravenous antimicrobial therapy (Table 4). Sepsis was observed in 18.9% (7/37) of patients, with blood cultures positive for *Klebsiella pneumoniae*, *Pseudomonas aeruginosa*, *Enterobacter hormaechei*, *Aspergillus flavus*, and *Aspergillus fumigatus*.

Importantly, no patient was admitted to the ICU due to severe infection or major hemorrhage, nor did any patient die during induction therapy.

### Univariate and multivariate analysis

Univariate and multivariate analyses were conducted to identify factors influencing OS. OS did not differ significantly based on WBC count, BM blast percentage, NPM1 mutation status, treatment response, or MRD status after the first cycle. However, patients with an ECOG ≥ 3 or a complex karyotype showed significantly worse OS. IDH1/2 mutations, receipt of allo-HSCT were identified as potential predictive factors associated with longer OS (Table 5).

**Table 5:** Univariate and multivariate analysis of OS

Variables	UVA			MVA	
	Hazard ratio (95% CI)	*p *value		Hazard ratio (95% CI)	*p *value
Age					
< 60		Ref		Ref	
≥ 60	1.14 (0.41–3.16)	0.81		0.11 (0.02–0.88)	< 0.05
ECOG					
< 3		Ref		Ref	
≥ 3	5.08 (1.68–15.4)	< 0.05		18.44 (2.16–157.33)	< 0.05
WBC count					
< 10 × 10E9/L		Ref		Ref	
≥ 10 × 10E9/L	0.60 (0.25–1.44)	0.26		0.36 (0.10–1.26)	0.11
BM blast					
< 50%		Ref	Ref		
≥ 50%	2.55 (0.92–7.06)	0.07	1.32 (0.31–5.71)		0.71
Karyotype					
Non-complex		Ref	Ref		
Complex	4.64 (1.52–14.2)	< 0.05	58.09 (5.74–588.31)		< 0.05
FLT3-ITD					
FLT3-ITD WT		Ref	Ref		
FLT3-ITD Mut	2.43 (0.95–6.20)	0.06	4.48 (0.95–21.11)		0.06
IDH1/2					
IDH1/2 WT		Ref	Ref		
IDH1/2 Mut	0.34 (0.08–1.47)	0.15	0.13 (0.02–0.86)		< 0.05
NPM1					
NPM1 WT		Ref	Ref		
NPM1 Mut	0.57 (0.17–1.98)	0.38	0.24 (0.04–1.44)		0.12
Response after the first cycle					
CR/CRi		Ref	Ref		
PR/NR	3.41 (1.34–8.66)	< 0.05	1.56 (0.16–15.05)		0.70
MRD after the first cycle					
Negativity		Ref	Ref		
Positivity	2.34 (0.96–5.74)	0.06	0.58 (0.05–6.47)		0.66
Received allogeneic HSCT					
Yse		Ref	Ref		
No	4.49 (0.60–33.70)	0.14	1327.76 (14.57–120964.82)		< 0.05

## Discussion

Advanced age has long been recognized as a factor associated with poorer prognosis in AML. The 5-year OS rate was reported to be 29%, but declined significantly to 8% in patients aged ≥ 65 years [19]. Previously, patients over 60 years of age were considered to have a higher prevalence of unfavorable genetic profiles, antecedent MDS, increased incidence of multidrug resistance, and more frequent comorbid conditions, collectively contributing to elevated treatment-related mortality [20]. In recent years, however, there has been a shift away from using age alone as a decisive factor [21]. The National Cancer Network currently recommended that treatment decisions should be guided by overall fitness rather than chronological age [20]. The younger patients with high-risk features may also be considered for less intensive induction regimens [20]. In our study, we assessed patients using clinical parameters such as age, comorbidities, and performance status. Ultimately, 37 patients were enrolled in our lower-intensity therapy cohort.

DNA methylation is an important therapeutic target in hematopoietic malignancies [22]. DNA demethylating agents, such as HMAs, restore the expression of tumor suppressor genes and are therefore widely used in the treatment of myeloid neoplasms [23]. Decitabine is one of the HMAs. At low dose, it induces epigenetic modifications, while at high doses it acts primarily as a cytotoxic agent [24]. Early studies employed DEC at high dose [25]. Subsequent investigations explored lower intravenous doses, ranging from 5 to 20 mg/m^2^ per day for 5–20 days per cycle [26]. Evidence suggested that these lower, epigenetically-targeted doses might be more effective against leukemia than higher, cytotoxic regimens [24]. In 2012, the European Medicines Agency approved DEC for the treatment of older or unfit patients with AML. However, the recommended dose of 20 mg/m^2^ once daily for 5 days remained intolerable for some elderly or debilitated patients. Later studies demonstrated the superior efficacy of a 10-day DEC regimen, particularly among patients with TP53 mutations [27]. More recently, alternative schedules have gained attention, including a low-dose weekly subcutaneous regimen (DEC 0.2 mg/kg every 7 days) and an oral combination of DEC/cedazuridine (35 mg decitabine daily for 5 days) [28]. Both of these regimens above were studied in combination with VEN and achieved CRc rates between 60 and 70%. In our study, patients received DEC at a dose of 6 mg/m^2^ intravenously for 10 days per cycle with VEN and the CRc rate was 80.5%.

Standard-dose of VEN-AZA is associated with prolonged cytopenia, an increased risk of febrile neutropenia, and a higher incidence of grade 3 infections [29]. For the frail patients with limited bone marrow reserves, reduced-intensity strategies are necessary. Studies revealed that shorter durations of VEN achieved comparable response rates while reducing hematological toxicity [17, 18, 30]. It is reported that there was no difference in CRc rate between a 14-day and a 28-day VEN regimen [17]. Similarly, comparisons among patients receiving VEN for 14, 21, or 28 days showed comparable CRc rates, ranging from 62 to 68% [30]. Another retrospective study indicated no differences in overall response or CRc rates between a 7-day VEN group and a standard VEN group (treatment lasting more than 14 days) [18]. However, some evidence suggested that reducing VEN exposure may not significantly alleviate cytopenia or lower infection rates [30]. n our study, VEN was administered for 14 days, which led to a reduction in cytopenia and related complications. Optimizing the duration of VEN therapy offers several potential benefits, including improved treatment compliance, decreased risk of drug interactions, lower medical costs, and the possibility for future exploration of triple-combination therapies.

We compared our results with those reported in the VIALE-A trial [5, 7]. The median follow-up duration in our study was shorter than that in VIALE-A (40.0 months vs. 43.2 months). Among patients who did not receive allo-HSCT, our cohort exhibited a longer median OS than VEN arm in VIALE-A (20.9 months vs. 14.7 months), along with a higher 24-month OS rate (53.1% vs. 37.5%). The median time to CRc was comparable between our study and the VEN arm in VIALE-A (1.4 months vs. 1.3 months), as was the median DOR among CR patients (30.2 months vs. 22.1 months). However, our CRc rate was higher (78.4% vs. 66.8%). Moreover, while 42% of patients in the VEN arm of VIALE-A achieved MRD response, our study showed a higher MRD negativity rate of 62.2% before the 2nd cycle. The median OS among patients who achieved MRD responses was similar between the two studies (34.4 months vs. 34.2 months). Additionally, our median EFS was longer than that of VIALE-A (18.8 months vs. 9.9 months). Overall, our regimen showed potential efficacy advantages over the treatment arm in VIALE-A trial. However, owing to the small sample size and lack of direct head-to-head comparison, additional studies are required for confirmation.

We further compared our outcomes against real-world datasets of HMA-VEN regimen [31, 32]. It was reported that the CRc rate was 64% in the United States and 64.8% in Thailand, whereas our cohort achieved a rate of 78.4%. Our intermediate/high risk patients exhibited a higher CRc rate (75.0% vs. 60% in US) according to the ELN 2022 risk stratification. In terms of OS, the 1-year OS rate in Thailand was 42.2%, and the 2-year OS rate in the US was 23%, while, our results showed a 1-year OS rate of 64.9% and a 2-year OS rate of 56.8%. Regarding relapse among responders, the rates was 59% in the US and 51.6% in Thailand, compared to our rate of 53.3%. Moreover, our DOR was 21.3 months, longer than the 3.8 months in Thailand. Notably, one patient relapsed after allo-HSCT but achieved CRi following treatment with DEC and VEN, and has survived for over 5 months to date.

We then compared grade ≥ 3 hematologic AE incidences across three cohorts: our regimen, the VEN arm of the VIALE-A trial, and real-world dataset [5, 32]. The incidence of neutropenia was 100% vs. 43% vs. 92.6%; thrombocytopenia was 73.3% vs. 46% vs. 75.9%; and anemia was 88.9% vs. 28% vs. 75.9%. The rates of hematologic toxicities in our cohort were higher than those in the VIALE-A, yet comparable to real-world clinical observations. Overall, our regimen was associated with more severe hematologic toxicity relative to the VEN arm of VIALE-A. Nevertheless, the majority of patients remained tolerable to our regimen. No AE-related deaths occurred during the induction phase, and hematologic toxicities were reversible with standard supportive care.

Patients with distinct molecular features exhibited heterogeneous prognoses. Despite suggestive prognostic relevance of FLT3-ITD, TP53, CBL and RUNX1::RUNX1T1 observed in our study, statistical significance was not attained. Consistent with prior study, TP53 mutation confers inferior clinical outcomes. In a cohort of 506 naive AML patients receiving HMA-VEN therapy, TP53 mutation was categorized as a high-risk genetic lesion associated with a median OS of merely 7.1 months [33]. The Mayo Genetic Risk Model also identified TP53 aberration as an adverse prognostic marker [34]. Moreover, TP53-mutant patients derive no definitive survival benefit from HMA-VEN compared with HMA [35]. In our study, four out of five TP53-mutant patients survived less than 7 months. FLT3-ITD is another poor prognostic factor. Together with NRAS and KRAS mutations, FLT3-ITD mutation acted as an independent adverse predictor in NPM1-mutated AML treated with HMA + VEN [36]. Our study demonstrated shorter median OS and EFS in FLT3-ITD-mutant patients (OS: 16.3 vs 48.2 months, EFS: 6.2 vs 31.5 months). CBL mutations frequently emerged as secondary mutations at disease relapse and represent an unfavorable biomarker for HMA-VEN therapy [33, 37]. Although the number of CBL-mutant cases in our data was small, mutational status tended to negatively impact both OS and EFS. Responses among different genetic mutation subgroups are of great clinical importance. However, owing to the limited sample size, we could only assess trends without detecting statistically significant disparities.

Our regimen showed a trend toward superior efficacy in patients with monocytic/mixed phenotypes, suggesting its potential to overcome the poor prognosis historically associated with HMA + VEN therapy in this subgroup [38].

Previous study has reported that when used as a bridge to allo-HSCT, HMA + VEN showed no significant differences in OS, relapse rate, or non-relapse mortality compared to the 3 + 7 regimen [39]. In our study, of the 5 patients who received allo-HSCT, 4 achieved CR and have remained in CR, with the longest current survival being 55.8 months. We propose that our regimen effectively reduced complications while eliminating malignant cells, thereby enabling patients to better tolerate allo-HSCT and achieve prolonged OS.

The main limitations of our study include its single-center, retrospective design, small sample size, and the absence of a control group. These factors may introduce confounding biases and limit the statistical power to detect significant differences. Furthermore, as IDH1/2 inhibitors were unavailable during the initial phase of the study, the outcomes may not fully align with the ELN 2024 risk stratification. To validate our findings, future research should involve large-scale, multicenter prospective clinical trials.

Our long-term retrospective study showed that the low dose DEC-VEN regimen had a high response rate and a favorable safety profile. This regimen may serve as a viable treatment option for patients with AML who are ineligible for intensive chemotherapy. Furthermore, it provides a backbone for future exploration of triple-agent combination therapies.

## Supplementary Information

Below is the link to the electronic supplementary material.

Supplementary file1 (PDF 604 KB)

Supplementary file2 (PDF 497 KB)

Supplementary file3 (PDF 181 KB)

## Data Availability

No datasets were generated or analysed during the current study.
