# Supplementary material for: Low-dose 10-day decitabine plus venetoclax induced rapid MRD negativity and durable remission in De Novo AML
Source: Ann Hematol. 2026 Aug 19;105(12):513. doi: 10.1007/s00277-026-07222-7 (PMC13768261; doi:10.1007/s00277-026-07222-7)

Supplmentary Figure 1

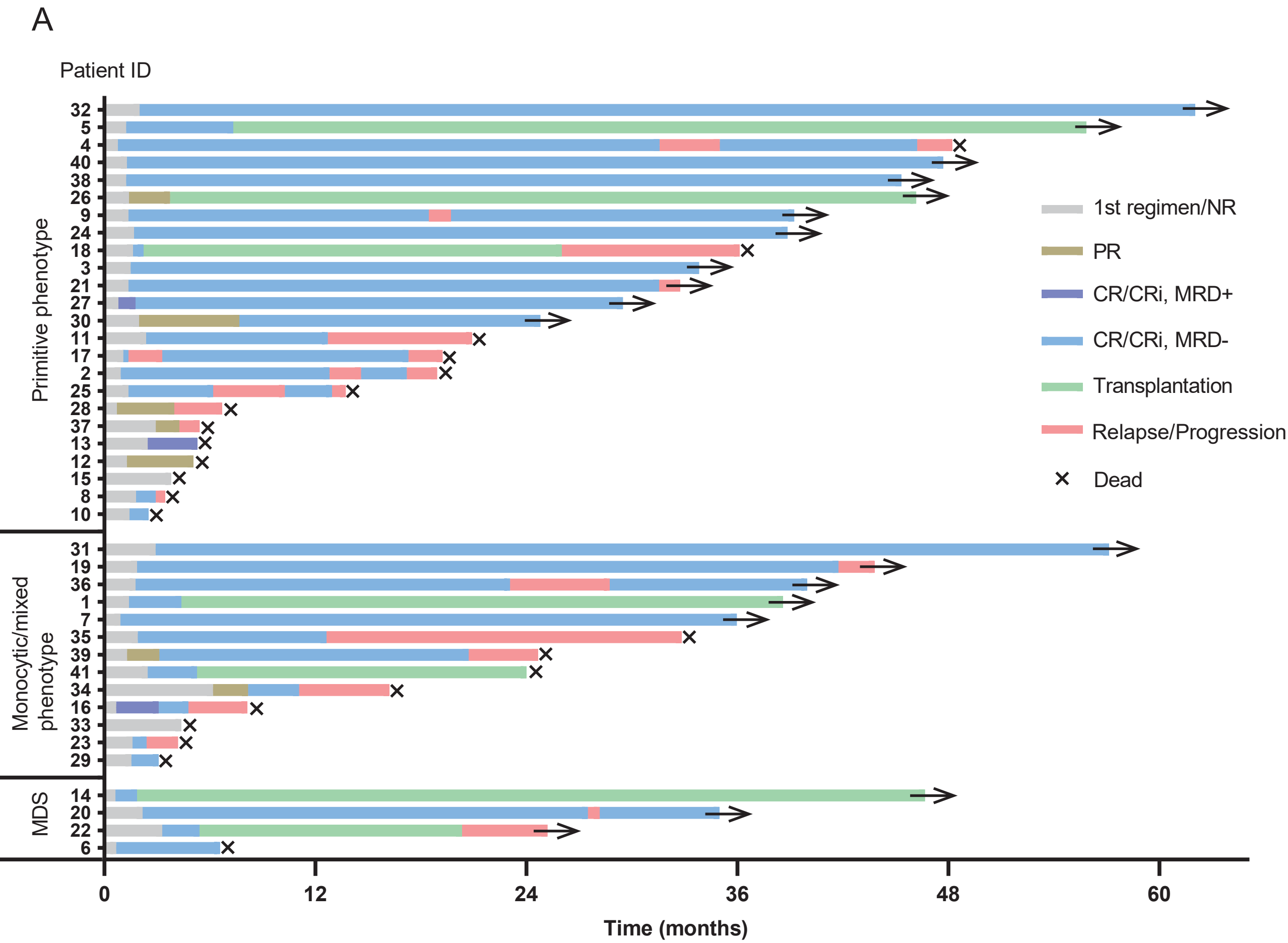

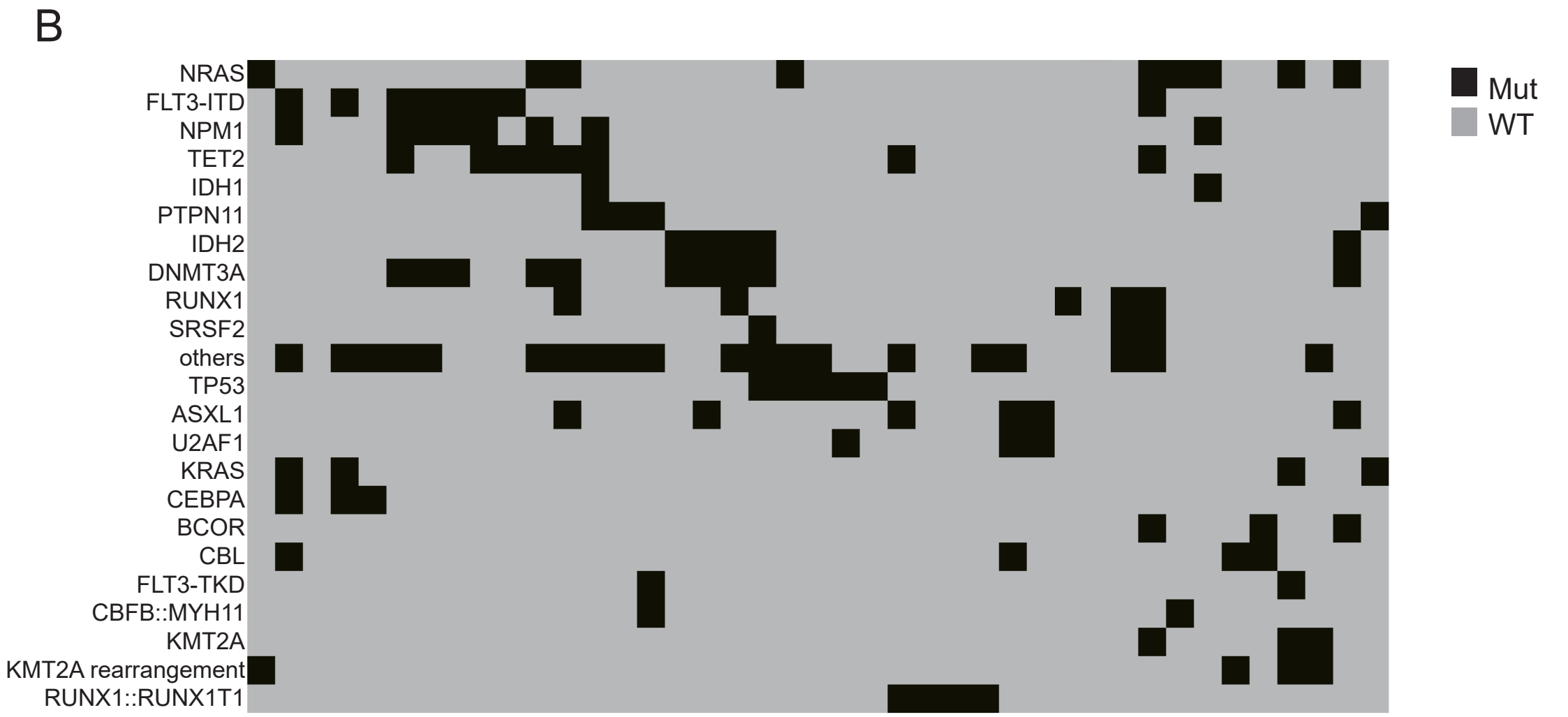

Supplement: Supplementary file 1 [file 277_2026_7222_MOESM1_ESM.pdf]
