# Supplementary material for: Low-dose 10-day decitabine plus venetoclax induced rapid MRD negativity and durable remission in De Novo AML
Source: Ann Hematol. 2026 Aug 19;105(12):513. doi: 10.1007/s00277-026-07222-7 (PMC13768261; doi:10.1007/s00277-026-07222-7)
Supplement: Supplementary file 2 [file 277_2026_7222_MOESM2_ESM.pdf]

Supplmentary Figure 2

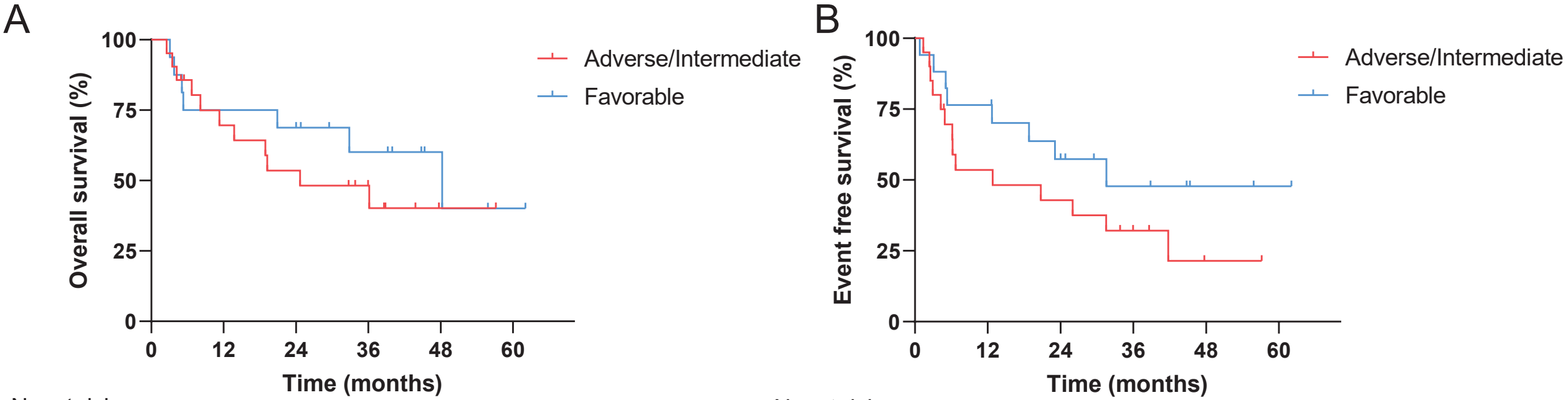

A No. at risk

No. at risk	0	12	24	36	48	60
Favorable	16	13	11	8	3	2
Adverse/ Intermediate	21	14	11	6	2	1

B No. at risk

No. at risk	0	12	24	36	48	60
Favorable	17	13	9	6	3	2
Adverse/ Intermediate	20	11	9	5	2	1
