# Supplementary material for: Low-dose 10-day decitabine plus venetoclax induced rapid MRD negativity and durable remission in De Novo AML
Source: Ann Hematol. 2026 Aug 19;105(12):513. doi: 10.1007/s00277-026-07222-7 (PMC13768261; doi:10.1007/s00277-026-07222-7)
Supplement: Supplementary file 3 [file 277_2026_7222_MOESM3_ESM.pdf]

Supplementary Table 1. Response assessment by phenotype

Variable	AML with primitive phenotype (n=24)		AML with monocytic/mixed phenotype (n=13)	
	n	%(95%CI)	n	%(95%CI)
**CRc rate**	17	70.8(51.2-90.4)	12	92.3(75.5-109.1)
CR	14	58.3(37.1-79.6)	10	76.9(50.4-103.4)
CRi	3	12.5(-1.8-26.8)	2	15.4(-7.3-38.1)
**CRc after the first cycle**	16	66.7(46.3-87.0)	10	76.9(50.4-103.4)
CR	13	54.2(32.7-75.7)	10	76.9(50.4-103.4)
CRi	3	12.5(-1.8-26.8)	0	0.0(0.0-0.0)
**MRD negativity after the first cycle**	15	62.5(41.6-83.4)	8	61.5(30.9-92.1)
**Partial remission after the first cycle**	4	16.7(0.6-32.7)	2	15.4(-7.3-38.1)
**No response after the first cycle**	4	16.7(0.6-32.7)	1	7.7(-9.1-24.5)
